# Optimal Reference Genes for Gene Expression Analysis of Overmating Stress-Induced Aging and Natural Aging in Male *Macrobrachium rosenbergii*

**DOI:** 10.3390/ijms26083465

**Published:** 2025-04-08

**Authors:** Yunpeng Fan, Qiang Gao, Haihua Cheng, Xilian Li, Yang Xu, Huwei Yuan, Xiudan Yuan, Songsong Bao, Chu Kuan, Haiqi Zhang

**Affiliations:** 1 Agriculture Ministry Key Laboratory of Healthy Freshwater Aquaculture, Key Laboratory of Fish Health and Nutrition of Zhejiang Province, Zhejiang Institute of Freshwater Fisheries, Huzhou 313001, China; yunpengfan1994@163.com (Y.F.); m130101022@163.com (H.C.); lixilian@126.com (X.L.); fionxu@foxmail.com (Y.X.); yuanhuwe@163.com (H.Y.); bss980702@163.com (S.B.); kuan_chu@163.com (C.K.); 2 School of Marine Science, Ningbo University, Ningbo 315211, China; 3 Hunan Fisheries Science Institute, Changsha 410153, China; yuanxd2024@163.com; 4 College of Life Science and Agri-forestry, Southwest University of Science and Technology, Mianyang 621010, China

**Keywords:** *Macrobrachium rosenbergii*, reference gene, overmating, aging, RT-qPCR, transcriptome

## Abstract

Functional gene expression is closely linked to an organism’s physiology and can be quantified using Real-Time Quantitative Reverse Transcription PCR (RT-qPCR). However, the stability of reference gene expression is not absolute, which may impact the accuracy of RT-qPCR results. In this study, we evaluated the suitability of nine genes including receptor for activated protein kinase c1 (*rack1*), ribosomal protein L6 (*rpl6*), ribosomal protein L9 (*rpl9*), ribosomal protein S2 (*rps2*), ribosomal protein S18 (*rps18*), ribosomal protein lateral stalk subunit P0 (*rplp0*), eukaryotic translation elongation factor 1β (*eef1b*), eukaryotic translation initiation factor 4a (*eif4a*), eukaryotic translation initiation factor 5a (*eif5a*) analyzed from RNA sequencing (RNA-Seq) data in addition to three genes including eukaryotic elongation factor 1α (*eef1a*), β-actin (*actb*), and glyceraldehyde 3-phosphate dehydrogenase (*gapdh*) selected from the literature to obtain the best internal controls in the RT-qPCR analysis of *M. rosenbergii* under overmating stress and natural aging. RefFinder was used to comprehensively evaluate the stability of the candidate reference genes. The initial results showed that three genes (*eif5a*, *rps18*, and *rplp0*) from the RNA-Seq data had relatively stable expression levels, which were more stable than those of the three commonly used reference genes. *Eif5a* and *rps18* were the best combination for the RT-qPCR analysis of *M. rosenbergii* under overmating stress and aging. Further analysis indicated that *eif5a* might be the best reference gene for the study of *M. rosenbergii.*

## 1. Introduction

In response to varying conditions and stimuli, the expression and regulation of functional genes in the tissues or cells of organisms exhibit significant variation [1,2]. This variation primarily involves the mechanisms of transcriptional and translational regulation [3,4]. Developing antibodies to detect proteins can be expensive, whereas measuring changes in transcript levels is more cost-effective and straightforward. As a result, transcript level analysis has become widely adopted [2,5]. Quantitative real-time PCR (qPCR) technology is a popular method for detecting gene transcription levels in various samples [1]. However, when comparing different samples, it is essential to standardize the transcript levels of the target genes [6,7]. In this context, the use of stably expressed reference genes as internal controls is crucial [8]. These reference genes, commonly known as housekeeping genes, include *β-actin* (*actb*) [9,10], eukaryotic elongation factor 1-α (*eef1a*) [7], and glyceraldehyde-3-phosphate dehydrogenase (*gapdh*) [11], amongst others. Nevertheless, it is important to note that no reference gene remains stable under all conditions, and their expression can vary depending on the tissue type, developmental stages, and experimental conditions [7,12]. Therefore, identifying the most stable reference genes for specific experimental conditions is essential.

Several reference genes have been screened and identified in various aquatic species, such as *Danio rerio* [13], *Carassius auratus*, *Cyprinus carpio* [14], *Xenopus laevis* [15], *Penaeus vannabinicus* [16], and *Macrobrachium nipponense* [7]. *M. rosenbergii*, freshwater shrimps with considerable economic value, is known for the significant growth advantages of the male individuals [17]. Research on *M. rosenbergii* spans multiple domains, including reproduction, nutrition, sex control, and disease [17,18,19,20]. In addition to stressors such as fighting, hypoxia, poor water quality, and bacterial and viral infections, male *M. rosenbergii* also experience senescence during normal breeding cycles. These aging males, nearing the end of their life span, exhibit a darkened body coloration, a red tail fan, reduced feeding, and slow movement. In practical breeding operations, the female-to-male ratio in the mating groups is typically greater for males at 3:1 [21,22,23]. Consequently, male attrition is often much higher than that of females after multiple breeding cycles, leading to an increased mating burden on the surviving males. Overmated males undergo noticeable changes, including a color shift from blue to orange, and their tails may even turn orange-red. While a few may survive for a short period after ceasing mating, most, resembling aged individuals, succumb soon thereafter. As mentioned above, compared to the normal male shrimp, these dying males have a lot in common. Therefore, this overmating behavior may lead to premature male senescence. Further study of the molecular regulation mechanism requires the detection of the gene transcription level. Generally, *eef1a*, *actb*, and *gapdh* are used as the reference genes in *M. rosenbergii* [11]. Nevertheless, the suitability of the reference genes can be affected by experimental conditions [7,12]. Because of this, it is essential to identify and validate the most stable internal reference genes across the three distinct physiological conditions in male *M. rosenbergii*.

In this study, four tissues and 36 transcriptome datasets from three types of male *M. rosenbergii* were used to screen candidate reference genes. Nine candidate reference genes were used (including receptor for activated protein kinase c1 (*rack1*), ribosomal protein L6 (*rpl6*), ribosomal protein L9 (*rpl9*), ribosomal protein S2 (*rps2*), ribosomal protein S18 (*rps18*), ribosomal protein lateral stalk subunit P0 (*rplp0*), eukaryotic translation elongation factor 1β (*eef1b*), eukaryotic translation initiation factor 4a (*eif4a*), eukaryotic translation initiation factor 5a (*eif5a*), and three reported reference genes (*eef1a*, *gapdh*, and *actb*). The Cq (Quantitative Cycle) values were analyzed using RefFinder [8,24], and the stability of the reference genes was assessed using geNorm [25], Normfinder [26], BestKeeper [27], and the comparative Δ-Ct method [28]. The results showed that the candidate genes were more stable than the commonly used reference genes and could provide better reference genes for the qRT-PCR standardized analysis of *M. rosenbergii* under mating stress.

## 2. Results

### 2.1. Analysis of Male M. rosenbergii in Three Different Physiological States

**Figure 1:**
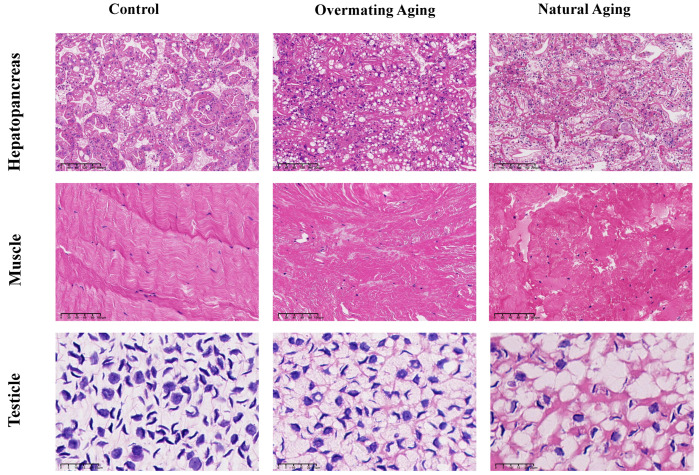
Histological sections of hepatopancreas, muscle, and testicle of male *M. rosenbergii* in three different physiological states.

**Figure 2:**
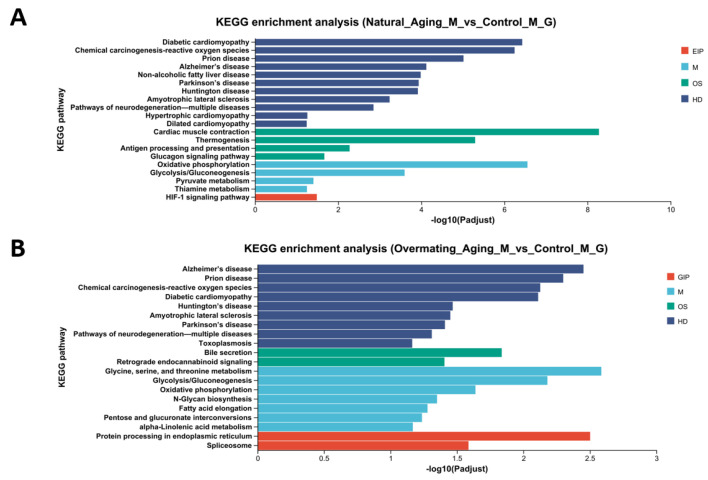
KEGG enrichment pathways of significantly different genes in muscle. (**A**) Natural Aging Group compared with Control Group. (**B**) Overmating Aging Group compared with Control Group.

**Figure 3:**
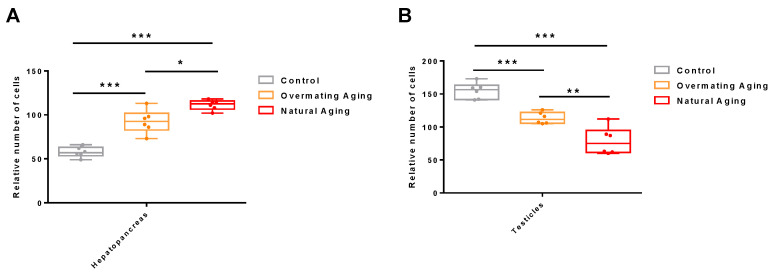
Statistical analysis was performed based on histological sections of hepatopancreas (40× microscope) and testes (80× microscope). (**A**) Relative number of hepatopancreatic cells; (**B**) relative number of spermatids. *: *p* < 0.05; **: *p* < 0.01; ***: *p* < 0.001.

To determine whether the two moribund states of *M. rosenbergii* were in an ‘ageing’ state, histological observations were made on their hepatopancreas and their muscle and testicle tissues (Figure 1), and differential gene enrichment analysis was performed on the muscle tissue (Figure 2). Hematoxylin and eosin (HE) staining revealed that both the liver and muscle tissues were severely damaged compared to those of the healthy male *M. rosenbergii*, characterized primarily by the destruction of the hepatopancreatic tubule structure, muscle fiber rupture, and blurred texture. Significant differences in the number of cell nuclei in the hepatopancreas were observed within the same field of view as follows: Control Group (57.67 ± 5.37) < Overmating Aging Group (92.50 ± 12.23) < Natural Aging Group (111.33 ± 5.22) (Figure 3A). Similarly, significant differences were found in the number of sperm within the same field of view as follows: Control Group (154.83 ± 11.04) > Overmating Aging Group (113.50 ± 8.06) > Natural Aging Group (78.83 ± 18.97) (Figure 3B). Significantly differentially expressed genes in the muscle tissue were enriched in age-related pathways, including Alzheimer’s disease, Parkinson’s disease, Huntington’s disease, amyotrophic lateral sclerosis, diabetic cardiomyopathy, chemical carcinogenesis-reactive oxygen species, and neurodegenerative pathways (multiple diseases). Together with other characteristics observed in the two types of *M. rosenbergii*, such as reduced feeding, decreased activity, and reduced molting frequency, it can be concluded that they are in an aging phase, with excessive mating contributing to the progression of aging in males.

### 2.2. Selection of Candidate Reference Genes

**Figure 4:**
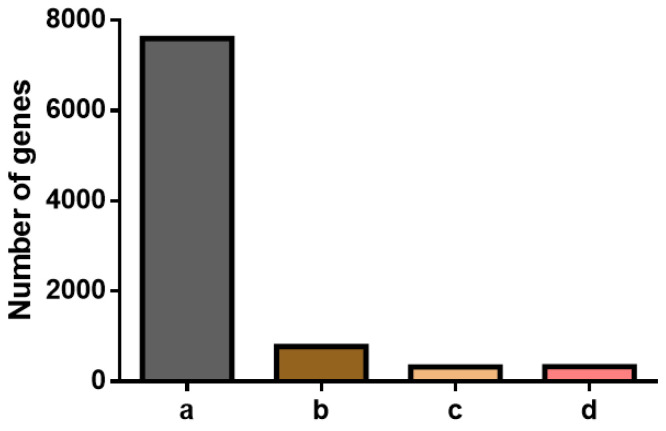
Gene numbers after applying the four screening criteria to *M. rosenbergii* transcriptome data: (a) TPM > 0; (b) standard deviation of log_2_(TPM) < 1; (c) the difference between log_2_(TPM) and the mean log_2_(TPM) less than two; (d) mean log_2_(TPM) > 5.

In 36 RNA-seq datasets, 3 candidate reference genes for male *M. rosenbergii* under different physiological conditions were selected from a total of 43,155 genes. Based on the four criteria mentioned above, 7598 (17.61%), 776 (1.80%), 320 (0.74%), and 328 (0.76%) genes were identified (Figure 4). The further estimation and selection of candidate reference genes were performed using the coefficient of variation (CV) values, ultimately identifying nine genes with a CV value less than 0.1 (Table 1). These candidate reference genes exhibited high expression levels, with an average log2(TPM) range from 6.02 to 9.20 and low CV values ranging from 0.062 to 0.099.

**Figure 5:**
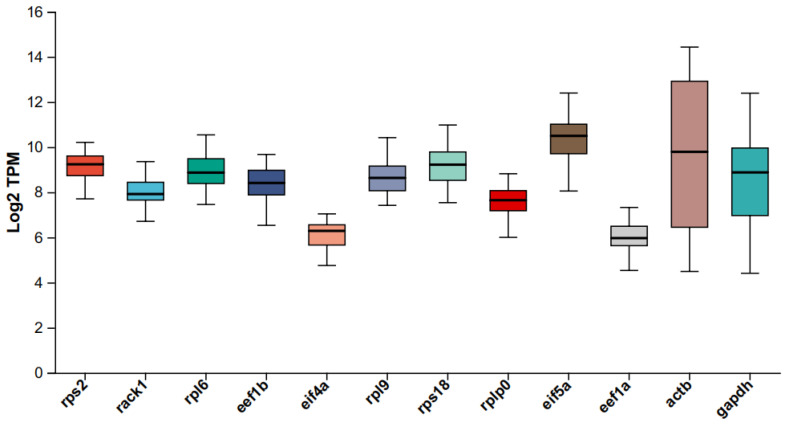
The expression stability of nine new candidate genes and three previously reported reference genes in *Macrobrachium rosenbergii* was assessed based on RNA-seq data. The boxplot illustrates the log2(TPM) values of these genes across 36 transcriptomes.

The log_2_(TPM) values from RNA-seq were utilized to assess the stability of the candidate genes (*rack1*, *rpl6*, *rpl9*, *rps2*, *rps18*, *rplp0*, *eef1b*, *eif4a*, and *eif5a*) and the reported reference genes (*eef1a*, *actb*, and *gapdh*). As demonstrated in Figure 5, *actb* exhibited the most significant variation, ranging from 4.49 to 14.43, while the *gapdh* gene demonstrated a substantial amount of variation, ranging from 4.41 to 12.39. The fluctuation of *eef1a* was minimal (4.53–7.32). In contrast, the log_2_(TPM) values of the candidate genes identified from the transcriptome exhibited considerably less variation across tissues, with *rack1* (6.19–9.35), *rpl6* (7.46–10.54), *rpl9* (7.42–10.42), and rps2 (7.71–10.21) displaying the most significant fluctuations. The remaining genes showed the following ranges: *rps18* (7.54–10.98), *rplp0* (5.82–8.82), *eef1b* (6.53–9.67), *eif4a* (4.75–7.04), and *eif5a* (7.59–12.4).

### 2.3. Distribution of Cycle Threshold (Quantitative Cycle) Values

**Figure 6:**
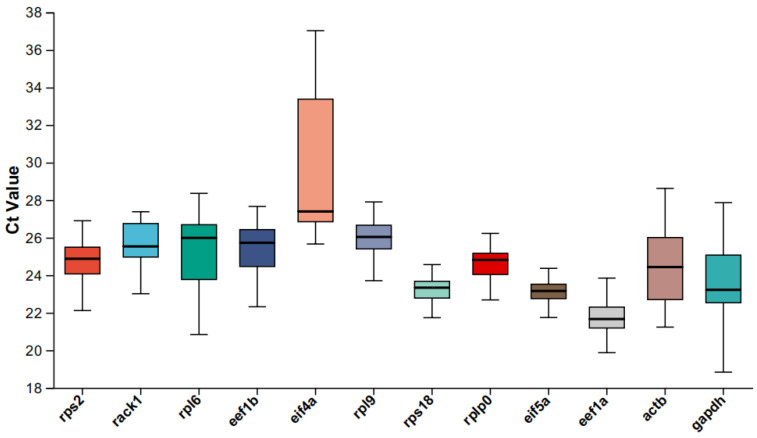
The expression stability of the new candidate genes and reported reference genes was evaluated based on the RT-qPCR results. The boxplot displays the Ct values of nine new candidate genes and three previously reported reference genes.

The qPCR primer pairs for the twelve genes are shown in Table 2. The correlation coefficient of the standard curve was > 0.98, and the amplification efficiency of the primers ranged from 94% to 104% (Table 2). The Ct values reflect the expression level of a gene, with lower values indicating higher abundance. The stability of gene expression can be inferred from the fluctuation range of the Ct values. As shown in Figure 6, the box plots demonstrate the quantitative expression levels of the candidate genes in different tissues. The vast majority of the candidate reference genes had Ct values ranging from 18 to 25 across different tissues, and their expression was stable in different tissues, including *rack1* (23.01–27.38), *rps2* (22.11–26.90), *rplp0* (22.34–26.22), *eef1b* (22.31–27.66), *rpl9* (23.70–27.90), *rps18* (21.73–25.29), *eif5a* (21.74–25.05), and *eef1a* (29.87–24.07). However, the expression levels of *gapdh* (18.83–27.86), *actb* (21.23–28.62), *rpl6* (20.84–28.36), and *eif4a* (25.66–27.02) fluctuated considerably.

**Figure 7:**
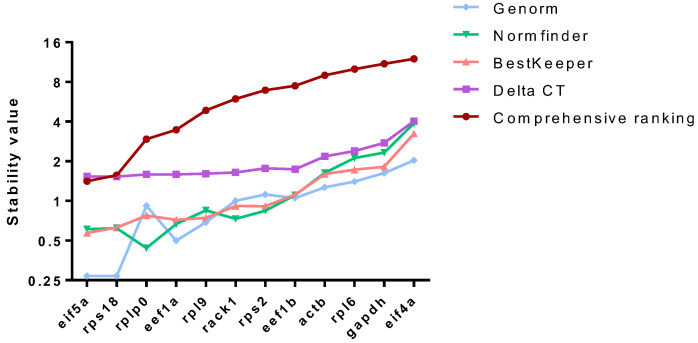
Based on the cycle threshold (Cq) data obtained from RT-qPCR, stability was evaluated using geNorm, NormFinder, BestKeeper, ΔCt, and a comprehensive ranking. Lower values indicate better gene stability.

The reference genes were evaluated and screened using the RefFinder program, which integrates geNorm, NormFinder, BestKeeper, and the comparative Δ-Ct methods and provides a comprehensive ranking by weighting (Figure 6). According to the NormFinder method, *rplp0* was identified as the optimal reference gene. GeNorm, BestKeeper, Δ-Ct, and comprehensive analysis identified *eif5a* and *rps18* as the most stable genes (Figure 7). GeNorm and comprehensive analysis identified these two genes as the most stable. We conclude that *eif5a* and *rps18* represent the optimal reference gene combination for aging-related studies of *M. rosenbergii*.

### 2.4. The Stability of eif5a and rps18 as Internal Controls Under Different Conditions Was Further Evaluated

**Figure 8:**
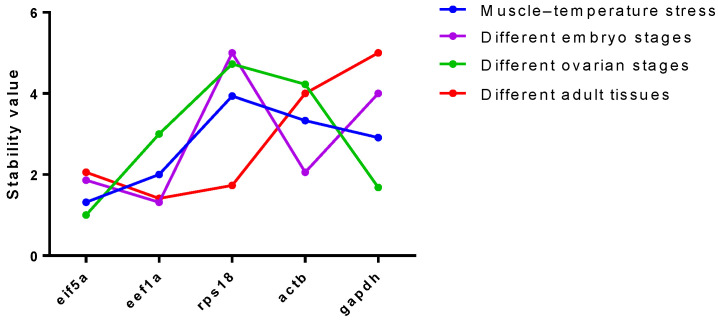
Comprehensive ranking of the reference genes under different conditions by RefFinder.

As shown in Figure 8, the reference genes were evaluated using RefFinder, where a lower score indicates a more stable gene. The best reference gene for muscle tissue under temperature stress is *eif5a*, which is also the most stable gene during ovarian development. The best reference gene is eef1a in embryonic development and different adult tissues, although *eif5a* is still relatively stable. The *rps18* gene was stable only in adult tissues. Comprehensive evaluation showed that eif5a was a relatively stable reference gene for *M. rosenbergii*.

## 3. Discussion

Quantitative real-time PCR (qPCR) is a routine method for gene transcription levels [1]. According to the China National Center for Bioinformation–National Genomics Data Center (CNCB-NGDC, https://ngdc.cncb.ac.cn/icg/gene/list, accessed on 8 December 2023), 345 genes are commonly used for internal RT-qPCR normalization [12]. The names of these genes appeared 2618 times in total, and the top three were actin (246, 9.40%), elongation factor (234, 8.94%), and glyceraldehyde-3-phosphate dehydrogenase (82, 6.95%). In previous studies on *M. rosenbergii*, the commonly used reference genes (*actb*, *eef1a*, and *gapdh*) were traditional reference genes [11,18]. However, no gene could be stable under all conditions. Therefore, it is crucial to select stably expressed reference genes when comparing different samples [8].

We identified male *M. rosenbergii* at the senescence stage, one of which was natural and the other due to overmating. Compared with normal male *M. rosenbergii*, their differentially expressed genes were significantly enriched in aging-related signaling pathways. Additionally, both types of aging showed similar tissue damage, including disrupted tight junctions in the hepatopancreatic cells, muscle fibrolysis, and reduced spermatogenic capacity in the testes. In this study, we used the methods of Li et al. [29] and Han et al. [30]. Nine candidate reference genes (*rack1*, *rpl6*, *rpl9*, *rps2*, *rps18*, *rplp0*, *eef1b*, *eif4a*, and *eif5a*) were selected based on 36 transcriptome datasets, which were widely and stably expressed in a variety of tissues with a low coefficient of variation (CV). Three traditional reference genes (actb, gapdh, and eef1a) were selected from previous studies. Four stability assessment methods—GeNorm [25], NormFinder [26], BestKeeper [27], and the Comparative ∆Ct method [28]—are commonly used for selecting reference genes in both animals [4,6,31,32] and plants [33,34]. The RefFinder program integrates these four methods to provide a comprehensive ranking [8]. We used the RefFinder software to evaluate the stability of these 12 genes based on the Ct values obtained by qRT-PCR. The stability ranking of the candidate genes was *eif5a* > *rps18* > *rplp0* > *eef1a* > *rpl9* > *rack1*> *rps2* > *eef1b* > *actb* > *rpl6* > *gapdh* > *eif4a*. The results obtained by the various methods in this study were generally similar, and the vast majority of the candidate reference genes screened from the RNA-Seq data were more stable than the conventional reference genes. The combination of *eif5a* and *rps18* was identified as the best reference gene combination for male *M. rosenbergii* under aging conditions. Eukaryotic translation initiation factors are crucial protein complexes that play a key role in initiating protein synthesis in eukaryotic organisms [35]. Among them, *eif5a* is widely distributed in archaea and eukaryotes and has remained highly conserved throughout evolution [36]. This factor is known to facilitate peptide chain elongation during the translation process and is typically present in high concentrations within cells [37]. The ribosomal protein S18, which is encoded by the *rps18* gene, is a critical component of the ribosome and plays an active role in protein synthesis [38]. Similarly to the present study, *rps18* shows high stability in the different life stages of bees [39].

Since the stability of the *eif5a* and *rps18* genes was significantly higher than that of other 10 reference genes, we further compared them with three traditional reference genes under different conditions. The *rps18* and *eef1a* genes were relatively stable, followed by *eif5a*, in the different tissues of male and female adults of *M. rosenbergii*. The health of gonadal development significantly impacts subsequent embryo development and juvenile weight, making it a critical focus of breeding programs [40]. The ovaries exhibit significant morphological and gene expression variations across different stages of development [41,42,43]. For the study of ovarian development regulation, *eif5a* and *gapdh* were identified as the most suitable reference genes at five distinct stages of ovarian development. The hatching of fertilized eggs is one of the key links in seedling cultivation, and the two most stable reference genes are *eif5a* and *eef1a* at the embryonic development stage. The muscle of *M. rosenbergii* is highly valued for its taste and nutritional content [18]. Faster growth and higher meat yield have long been key traits in breeding programs [17,44]. Temperature is an important environmental factor that affects growth, and the regulation of genes related to muscle growth changes under temperature stress [45,46]. The results of yjr temperature stress experiment showed that the expression level of *eif5a* in muscle tissue remained highly stable.

In summary, we conducted a comprehensive evaluation of twelve candidate reference genes using RefFinder. Based on the results presented in this study, we recommend the combination of *eif5a*, *rps18*, and *ef1a* for most experimental studies involving *M. rosenbergii* and advise avoiding *gapdh* and *actb* as reference genes whenever possible. The reference genes identified in this study will serve as valuable resources for gene expression analysis in *M. rosenbergii*.

## 4. Materials and Methods

### 4.1. Animals and Experiment Design

*M. rosenbergii* were hatched in April 2023 in Changxing, China. After approximately 13 months of rearing, individuals with an average size of 14.7 ± 0.6 cm were obtained. The samples consisted of male *M. rosenbergii* in three distinct physiological states, with each group comprising six biological replicates. The groups were classified according to the following criteria:

Type 1 (Control Group): Individuals exhibited a light-bluish–blue coloration, a shiny exoskeleton, high reactivity, and normal feeding behavior.

Type 2 (Overmating Aging Group): The female *M. rosenbergii* with ovaries full of the entire cephalothorax were put in and mated with the male. After the female had eggs, the female with eggs was transferred, and new females were added to achieve the purpose of frequent mating. After 30 to 40 mating times, these individuals showed an orangey-blue body color due to the frequent mating, prolonged guarding of females, reduced foraging activity, and shell fading frequency; a less bright exoskeleton color; and even an orange-red tail fan in some cases.

Type 3 (Natural Aging Group): The outer shell was dark, hard, and dull; the head and chest were dark red; and the tail fan was red. Decreased food intake and slow movement were observed. Death usually occurred within a few days of detection.

**Figure A1:**
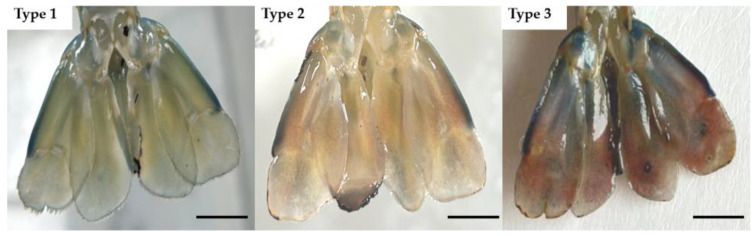
The tail fan characteristics of three types of *M. rosenbergii*. Scale bar = 1 cm.

The tail fan characteristics of the above three types of prawns are shown in Figure A1.

For the temperature treatment experiment, three groups of 30 shrimp each were kept at 18 °C, 25 °C, and 32 °C for 7 days. At least three biological replicates were guaranteed for all the experimental samples.

Adult *M. rosenbergii* (10.0 ± 0.5 cm) were sampled in the following order: eyestalk, gill, heart, hepatopancreas, gonads (ovary, testis), and finally muscle. According to the state of oocyte development, the ovary was divided into five stages. Embryo samples at eight different stages were collected based on the characteristics of embryo development.

### 4.2. Histological Observation

Hematoxylin and eosin staining of the muscle tissue was performed. Briefly, tissues were washed thrice with Phosphate Buffer Saline, fixed in Bouin’s solution for less than 24 h, transferred to 4% paraformaldehyde, embedded in paraffin, cut into 4 µm sections, and then stained with hematoxylin and eosin (H&E). The histological changes in muscle tissue were observed by microscopy. The relative number of hepatopancreas and spermatid cells was calculated according to the number of nuclei, and data were obtained from six different views of histological sections for each group.

### 4.3. RNA Extraction, Sequencing, and Synthesizing cDNA

Immediately after tissue samples were collected, they were frozen in liquid nitrogen and subsequently transferred to an ultra-low-temperature freezer at −80 °C for storage. Samples were thoroughly ground in the presence of added liquid nitrogen, and total RNA was extracted using the RNA isolator total RNA extraction reagent (Vazyme Biotech Co., Ltd., Nanjing, China). RNA-Seq was performed on *M. rosenbergii* carapace from different groups from Shanghai Meiji Biomedical Technology Co., Ltd., Shanghai, China. Each sample was tested three times. In total, 1 µg RNA was reverse-transcribed using the HiScript III 1st Strand cDNA Synthesis Kit (+gDNA wiper) (Vazyme Biotech Co., Ltd., Nanjing, China). cDNA was diluted to a 10-fold concentration and subsequently stored at −40 °C.

### 4.4. Selection of Candidate Reference Genes from Transcriptome Data and Primer Design

**Figure A2:**
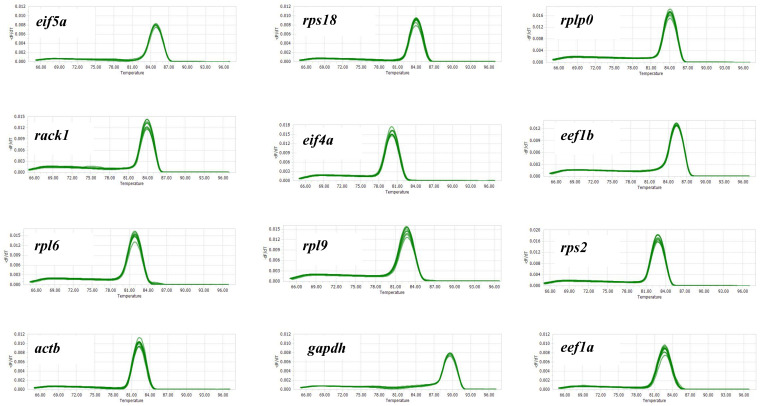
The amplification product of each primer pair that yielded a single melting peak.

We followed the criteria described by Li et al. [29] and Han et al. [30] to identify candidate reference genes. Four standards were employed for the selection of reference genes: (I) the gene expression being detectable in muscle, hepatopancreas, testes, and heart tissues; (II) low tissue variance, with a standard deviation of log_2_(TPM) < 1; (III) the difference between the log_2_(TPM) of each sample and the mean log_2_(TPM) being less than 2; (IV) relatively high gene expression, with an average log_2_(TPM) > 5. Additionally, the stability of the reference genes was further evaluated using the coefficient of variation (CV) value (stdev/mean). In this study, the CV values of the candidate reference genes screened from the transcriptome were all less than 0.1. The primer design followed MIQE guidelines, and the amplification product of each primer pair yielded a single melting peak (Figure A2), reflecting its stability and specificity.

### 4.5. Real-Time Quantitative PCR (qPCR) Analysis of Gene Expression

The qPCR in this study followed the MIQE guidelines (Minimum Information for Publication of Quantitative Real-Time PCR Experiments) [47]. The total qPCR reaction system comprised 10 µL, containing 1 µL of cDNA at a concentration of 5 ng/µL, 0.5 µL of each complementary pair of primers, and 5 µL of 2 × ChamQ Blue Universal SYBR qPCR Master Mix (Vazyme Biotech Co., Ltd., Nanjing, China). For each sample, experiments were repeated three times. The aforementioned reaction liquids were added to 0.1 mL of a 96-well plate (NEST Biotechnology Co., Ltd., Wuxi, China) under a low-light strip, and the qPCR reaction was performed after brief centrifugation. The reaction was conducted using a fluorescent quantitative PCR system (Roche LightCycler 96, Roche, Switzerland), with the following conditions: 95 °C for 2 min, followed by 95 °C for 10 s and 60 °C for 30 s, for a total of 45 cycles. The 2^−△△Ct^ method was used to calculate the relative expression ratio of the target genes to the reference genes [48].

### 4.6. Stability Assessment of Reference Genes

The qPCR data were collated to obtain the Ct values of the candidate internal reference genes in different samples of *M. rosenbergii*, and the stability of the expression of each gene was analyzed separately using the RefFinder online platform, an algorithm that combines four major computational methods, including geNorm, Normfinder, BestKeeper, and the Δ-Ct comparison method. Based on the rankings provided by the different programs, a relatively scientific overall ranking was derived after weighting [8].

## Figures and Tables

**Table 1:** Detail information on nine selected candidate reference genes and three commonly used reference genes.

Gene Name	Description	Mean	Stdev	CV Value
*rps2*	ribosomal protein S2	9.20	0.57	0.062
*rack1*	receptor for activated protein kinase c1	7.99	0.65	0.081
*rpl6*	ribosomal protein L6	8.94	0.73	0.082
*eef1b*	eukaryotic translation elongation factor 1β	8.41	0.71	0.084
*eif4a*	eukaryotic translation initiation factor 4a	6.11	0.53	0.087
*rpl9*	ribosomal protein L9	8.62	0.75	0.087
*rps18*	ribosomal protein S18	9.17	0.87	0.095
*rplp0*	ribosomal protein lateral stalk subunit P0	7.57	0.72	0.095
*eif5a*	eukaryotic translation initiation factor 5a	6.02	0.60	0.099
*eef1a*	eukaryotic elongation factor 1α	10.32	1.06	0.103
*actb*	β-actin	9.77	3.42	0.350
*gapdh*	glyceraldehyde-3-phosphate dehydrogenase	8.55	2.10	0.246

**Table 2:** Primer pairs for qPCR of candidate reference genes.

Gene	Primer Sequence (5′–3′)	Length (bp)	PCR Efficiency (%)	Correlation Coefficient
	Forward/Reverse			
*rps18*	TACCTACGACCCACACCCTT	137	98.0	0.99
	TATCAACGCACCGCCAAGAT			
*rack1*	TGTTTGGCCATCTGCAGACC	177	97.5	1.00
	CTTTGCTTCAGTCCCAACCG			
*rps2*	GCACGTGTCTGTTTCTGGAC	176	97.5	1.00
	TGGGTGCCAATCACCAAACT			
*rplp0*	ACATGTTGAGAAGCGTGGCT	163	96.0	1.00
	CCTGCCCAGAACACTGGATT			
*eif4a*	TCACCACAGACTTGCTTGCT	118	95.0	1.00
	AAACGTCCACCACGTCCAAT			
*eef1b*	GGCACTTTTCTTTGCAGCGT	178	94.0	1.00
	AGCCGAGAAGTCCAAGTTCC			
*rpl6*	CAAGTACGCACGAAGAAGCG	107	96.5	1.00
	AAAGACTTGCGGAGAGGAGC			
*rpl9*	AAACCGCAAGGAGGTAGCTG	106	98.0	1.00
	AGCGTATACAGCACGCATCT			
*eif5a*	CATGGATGTACCTGTAGTGAAAC	179	104.0	0.98
	CTGTCAGCAGAAGGTCCTCATTA			
*actb*	ACCACCATGTACCCAGGAATCGCTG	133	101.5	0.98
	CCAAGATTGAACCGCCGATCCAG			
*eef1a*	ACTGCGCTGTGTTGATTGTAGCTG	90	101.5	1.00
	ACAACAGTACGTGTTCACGGGTCT			
*gapdh*	TGAAGCCCGAGAACATTCCATG	170	104.0	1.00
	GTTCACGCCGCAGACGAACATG			

## Data Availability

All data directly related to this paper can be found in the body, Appendix A, or National Center for Biotechnology Information database. The raw rna-seq data can be accessed from the following url: https://www.ncbi.nlm.nih.gov/sra/PRJNA1239913, SRA ID: SRR32798172-SRR32798207.

## Abbreviations

*M. rosenbergii*	*Macrobrachium rosenbergii*
RT-qPCR	Real-Time Quantitative Reverse Transcription PCR
Ct	Quantitative Cycle/cycle threshold
*rps2*	Ribosomal protein S2
*rack1*	Receptor for activated protein kinase c1
*rpl6*	Ribosomal protein L6
*eef1b*	Eukaryotic translation elongation factor 1β
*eif4a*	Eukaryotic translation initiation factor 4a
*rpl9*	Ribosomal protein L9
*rps18*	Ribosomal protein S18
*rplp0*	Ribosomal protein lateral stalk subunit P0
*eif5a*	Eukaryotic translation initiation factor 5a
*eef1a*	Eukaryotic elongation factor 1α
*actb*	β-actin
*gapdh*	Glyceraldehyde-3-phosphate dehydrogenase
TPM	Transcripts per million
MIQE	Minimum Information for Publication of Quantitative Real-Time PCR Experiments
